# Implementing the FAIR Data Principles in precision oncology: review of supporting initiatives

**DOI:** 10.1093/bib/bbz044

**Published:** 2019-06-29

**Authors:** Charles Vesteghem, Rasmus Froberg Brøndum, Mads Sønderkær, Mia Sommer, Alexander Schmitz, Julie Støve Bødker, Karen Dybkær, Tarec Christoffer El-Galaly, Martin Bøgsted

**Affiliations:** 1 Department of Clinical Medicine, Aalborg University, Denmark; 2 Department of Haematology, Aalborg University Hospital, Denmark; 3 Clinical Cancer Research Center, Aalborg University Hospital, Denmark

**Keywords:** FAIR Data Principles, genomics, precision oncology, standards, data sharing

## Abstract

Compelling research has recently shown that cancer is so heterogeneous that single research centres cannot produce enough data to fit prognostic and predictive models of sufficient accuracy. Data sharing in precision oncology is therefore of utmost importance. The Findable, Accessible, Interoperable and Reusable (FAIR) Data Principles have been developed to define good practices in data sharing. Motivated by the ambition of applying the FAIR Data Principles to our own clinical precision oncology implementations and research, we have performed a systematic literature review of potentially relevant initiatives. For clinical data, we suggest using the Genomic Data Commons model as a reference as it provides a field-tested and well-documented solution. Regarding classification of diagnosis, morphology and topography and drugs, we chose to follow the World Health Organization standards, i.e. ICD10, ICD-O-3 and Anatomical Therapeutic Chemical classifications, respectively. For the bioinformatics pipeline, the Genome Analysis ToolKit Best Practices using Docker containers offer a coherent solution and have therefore been selected. Regarding the naming of variants, we follow the Human Genome Variation Society's standard. For the IT infrastructure, we have built a centralized solution to participate in data sharing through federated solutions such as the Beacon Networks.

## Introduction

The treatment of cancer has made significant progress in the past decades [1–4], but there are still too many patients who do not respond to treatment. The goal of precision medicine is to take a detailed view of each patient and their cancer, especially at the genomic level, to tailor their treatment accordingly. Genomic data throughout this article is defined as information on genes and gene expression. The genomic approach has revealed that most cancers are very heterogeneous [5–7], which implies that building prognostic and predictive models of sufficient accuracy requires a large quantity of data that is difficult to produce for any single research centre. This fact causes an evident need for data sharing to gather and analyse enough data to train complex models and uncover elusive patterns [8, 9].

Sharing data between research groups is not a challenge specific to health science but a widespread issue in research, resulting in the development of the Findable, Accessible, Interoperable and Reusable (FAIR) Data Principles [10], which define good data stewardship practices. The term `Findable’ implies data can be found online, typically through indexing in search engines. `Accessible’ means data can be retrieved directly or via an approval process. `Interoperable’ imposes data to follow standards. Finally, `Reusable’ requires the context of the data generation (metadata) is documented so it can be compared to or integrated with other data sets. These principles, initially developed for the academic world, are becoming a reference both at state [11] and industry [12] levels. Following these principles requires an application of standards to the various aspects of data collection and sharing.

However, large-scale data sharing in health science in general and in precision oncology in particular faces specific challenges [13, 14]. Leaving aside privacy and ethical issues, some of the major challenges lie in the ways data are recorded and stored. Various local and national health care systems and reporting traditions are often incompatible, making it complicated, expensive and time-consuming to aggregate data from different sources due to the amount of data management involved. Various initiatives have been launched to tackle these issues by standardizing and facilitating the implementation of data pipelines.

To support a local precision oncology project (registration number from the Danish National Committee on Health Research Ethics is N-20160089), we are developing a dedicated platform to collect, enrich and share clinical and genomic data. With the objective of implementing the FAIR principles in this platform, we will evaluate a selection of the aforementioned initiatives to see how they could make our solution FAIR regarding complexity and costs as well as ethical and legal aspects. The focus will be on the initiatives related to clinical and genomic data, as linking these two types of data is the most common and mature approach [15–18] to precision oncology.

Through a systematic literature review, we will first investigate initiatives that can support interoperability and reusability aspects in clinical and genomic data collection. Then, we will explore options and good practices to make data findable and accessible.

## Methods

Our 1st goal was to obtain an overview of the most recent initiatives centred on data sharing in precision oncology. To that end, we conducted a systematic literature review on PubMed, Scopus and Web of Science taking into account the PRISMA guidelines [19]. We used the following search criteria (for more details, see Table 1): (“cancer” AND “precision medicine” AND “data sharing”) OR (“genomics” AND “data sharing”).

**Table 1:** Literature review search queries

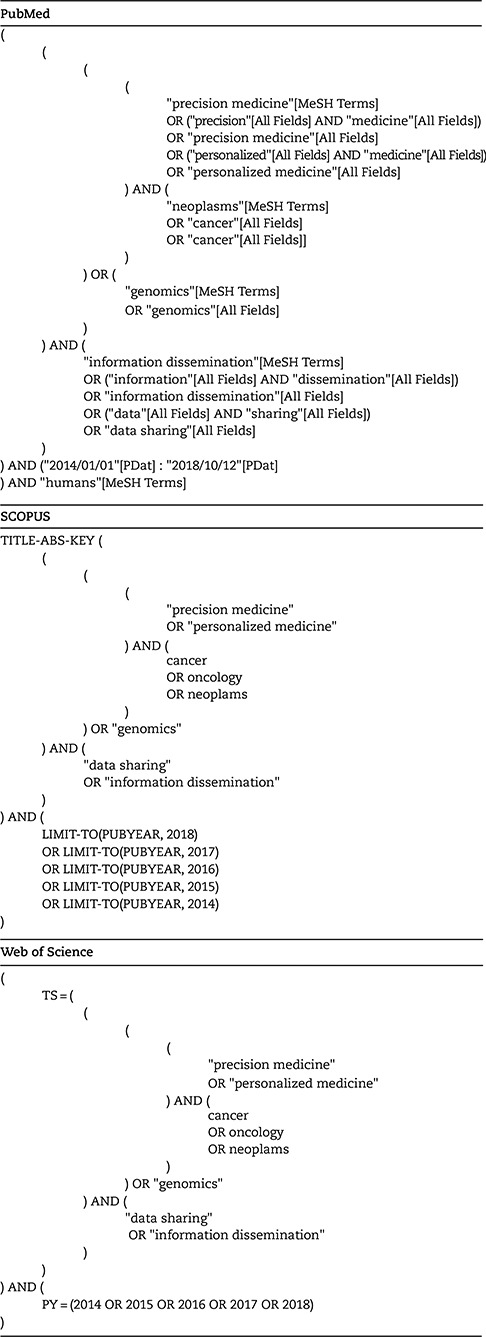

In this rapidly changing field, we decided to focus on the past 5 years (January 2014–October 2018) for our search. We also decided to focus on practical implementation and disregard legal and/or ethics and/or privacy and/or policy issues, which have been covered in other publications [20–24]. The inclusion criteria were the following:

- Directly applicable in the cancer context
- Directly applicable for data sharing
- Directly applicable to clinical and/or genomic data
- Not mainly treating legal and/or ethics and/or privacy and/or policy issues

**Figure 1:**
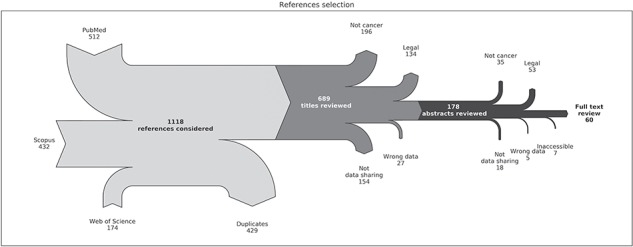
Flow chart of the reference selection process. The light grey part describes the collection of references from PubMed, Scopus and Web of Science and the removal of duplicate entries. The dark grey part describes the filtering of references based on titles. The black part describes the filtering of references based on abstracts. `Not cancer’ means `Not directly applicable to cancer’, `Legal’ means `Treating mainly legal and/or ethics and/or privacy and/or policy issues’, `Not data sharing’ means `Not directly applicable to data sharing’ and `Wrong data’ means `Not directly applicable to clinical and/or genomic data in human’. `Inaccessible’ refers to references where the full content could not be accessed or were not in English, Danish or French.

We found 1118 references among which 429 were duplicates (Figure 1). From the 689 unique references, we filtered out 411 using the criteria above based on titles.

From the 178 remaining references, we further filtered out 118 after review of the abstracts according to the criteria. A full-text review was then performed on 60 references.

## Results

### Clinical data

The clinical data encompass the information about patient status and disease phenotype. The patient status includes, for example, demographic information such as age and gender, medication, comorbidities, exposures, blood test results and treatment information. The disease phenotype is characterized by morphology and topography. The morphology details the cellular structure of the cancer, whereas the topography defines its location. Usually, these data are collected by healthcare personnel and stored in electronic health records (EHRs) or in a research and clinical trials context, as case report forms (CRFs), EHRs being defined as all healthcare data available about the patient in an electronic format.

### Data structure models

To support interoperability and reusability of data, the structure of the collected data must be consistent with other widespread data collection and storage solutions.

Several European countries have been working on EHRs for several years [25]. Denmark, for example, has largely solved the problem at a regional level but is still facing interoperability issues at the national level [26]. Large efforts are needed to converge to a more broadly accepted open standard.

The Fast Healthcare Interoperability Resources (FHIR) [27] standard has been designed to tackle the interoperability problem by specifying an interface for data exchange. This standard is related to another older initiative from Health Level Seven, the Clinical Document Architecture [28], focusing exclusively on clinical data. Due to its specificity, this format might be cumbersome outside of an EHR context. Furthermore, the format supports a multitude of data types but does not provide guidance on what to share.

At the CRF level, Clinical Data Acquisition Standards Harmonization [29] specifies data needed to be collected in a clinical trial to follow FDA (the US Food and Drug Administration) standards. This solution focuses on adverse events reporting and might only be adequate for clinical trials.

To find more appropriate formats, search engines make it possible to find alternatives. FAIRsharing.org [10], for example, aggregates content from other data and metadata specification repositories, such as medical data models [30]. The main issue with this type of solution is that the number of options is overwhelming, and it becomes difficult to find the needle in the haystack.

There are some tools to design a CRF following good practices, such as Centre for Expanded Data Annotation and Retrieval [31]. The Research Electronic Data Capture (REDCap) [32] solution also allows one to design CRFs, but the emphasis is more on implementation than on good practices, as it is not designed for specifications but for actual data collection. The problem remains that these solutions do not provide a clear guideline on what to collect in the context of data sharing.

Looking at actual data-sharing projects, the Genomic Data Commons (GDC) [33, 34] is a major resource. The GDC was launched in June 2016, and its goal is to share linked clinical and genomic data from the Therapeutically Applicable Research to Generate Effective Treatments [35] (TARGET) and The Cancer Genome Atlas (TCGA) [36] projects. This is the largest public data repository to date linking these two types of data. A major accomplishment of the GDC was its ability to successfully gather and share data from disparate sources in a harmonized way as detailed harmonization requirements and procedures were designed for that purpose. Notably, the GDC defines a list of data and metadata to link clinical and genomic data.

Disregarding issues caused by merging disparate data, the GDC data structure can be considered the *de facto* standard and therefore a logical choice for structuring data collection. For the actual data collection, REDCap is an excellent resource due to its flexibility and open API, allowing it to be easily integrated with existing solutions.

### Ontologies

The goal of ontologies is to ensure that the terms used are unambiguous and capable of describing concepts and relationships in an appropriate way. For example, the `lower limb’ medical phrase should have a clear definition and a relationship to the `foot’ phrase. Ontologies are essential to interoperability and shall ideally define languages understandable by both humans and machines.

The GDC project uses the simple ontology Cancer Data Standards Registry and Repository [37] (CaDSR) developed by the National Cancer Institute (NCI), which builds upon the common data elements (CDEs) to define data and metadata. Other more complex solutions, such as Logical Observation Identifiers Names and Codes [38] (LOINC) or Ontology for Biomedical Investigation, also attempt to tackle the definition and unambiguity issues [39].

To make ontologies more easily accessible, European Molecular Biology Laboratory–European Bioinformatics Institute, UK, has developed a search engine project called Ontology Lookup Service.

In an effort to standardize the naming of concepts, Systematized Nomenclature of Medicine—Clinical Terms (SNOMED CT) was developed and it is now a globally accepted nomenclature. It is notably collaborating with LOINC [40].

Nevertheless, ontologies may have complicated structures, which also make them harder to implement. Even SNOMED CT, which has a limited level of abstraction, is challenging to put in place [26].

In the context of oncology, precise classifications have been developed over the years to cover most of the characterization needs in the field. This situation limits the interest of implementing a complex ontology. Therefore, the CaDSR's CDE is an appropriate starting point as it follows a simple and pragmatic approach and it is implemented on the GDC platform.

### Classifications

Classifications are similar to ontologies as they define a common language, but they are much narrower in terms of scope, which makes their implementation straightforward. Moreover, there has been a much stronger movement towards convergence regarding classifications than ontologies. The observance of classifications is a mandatory requirement for the interoperability and reusability of collected data.

Most of this convergence was made possible through the World Health Organization (WHO), which is piloting major classification projects, including the International Classification of Diseases [41] (ICDs), International Classification of Diseases for Oncology [42] (ICD-O) and Anatomical Therapeutic Chemical [43] (ATC) classifications.

These classifications are widely used in EHRs, and even though countries, such as USA or Denmark, have deployed customized versions of ICD version 10, these customizations are limited, guaranteeing a high level of compatibility.

Adverse events can be classified using the Common Terminology Criteria for Adverse Events [44], but it is seldom reported in EHRs, hindering the usage of such data.

Using these standards is a step towards interoperability and improves findability because classifications facilitate search mechanisms.

Due to the global acceptance of the classifications listed above, ICD-0-3, ICD-10 and ATC are necessary and should be implemented as early as possible in the data collection process.

## Genomic data

Contrary to clinical data that can be stored and shared directly upon collection, a genomic data analysis starts with biospecimens of various origins (biopsy, blood, bone marrow, etc.) from which DNA or RNA is extracted. The genomic data are then generated from this material and often require further bioinformatics processing before it can be interpreted. The entire workflow needs to be standardized and documented to guarantee interoperability and reusability.

We are working with whole exome and RNA sequencing. We will thus focus on related solutions, but similar resources can be found for other high-dimensional data, such as pharmacogenomics [45,46] or imaging [47].

### Metadata requirements

The purpose of metadata is to document the workflow behind the produced data. The idea is that potential biases in a specific workflow can be identified and considered, which makes the metadata necessary for reusability. This method also promotes the standardization of workflows and thus interoperability.

Following the idea of sharing information about genomic data generation and in addition to other omics metadata initiatives [48], Minimum INformation about a high-throughput SEQuencing Experiment [49] (MINSEQE), by the Functional Genomics Data Society (FGED), define a minimum set of metadata for high-throughput sequencing (HTS), respectively, to guarantee the quality, documentation and reproducibility of the experiments.

The GDC requires one to provide a specific set of metadata, mostly overlapping with the FGED's requirements, to contribute to the platform. These requirements come from the data generated for the TARGET and TCGA projects. These projects are not very recent, so some aspects of these requirements might be outdated, but they still represent good practice.

The FGED's requirements have become standard, are used by major data-sharing platforms and thus should be followed.

### Processing standards

Raw files from microarrays and HTS are not directly usable, as they need to be processed. To make interoperability and reusability possible, processing should be performed in a standardized manner.

Numerous processing scripts and tools have been developed over the years by a multitude of centres. Initiatives such as bio.tools [50] aim to store these resources in a common repository and make them available through a search engine. The main goal is reusability to help researchers adapt existing solutions to their problems but also interoperability to make software used in a study openly available.

Moreover, there is a large trend towards containerization, with Docker being the leading solution [51,52].

The general idea is to have the entire environment packaged, including operating system, libraries, tools and scripts, instead of having metadata only specifying a limited part of the environment along the scripts. Docker containers can thus be easily deployed to other machines supporting Docker. Regarding reusability and interoperability, this is a game changer, eliminating the hassle and hurdles to reconfigure your environment to be able to run other projects [53].

This technology is also promoted by large consortia such as the Global Alliance for Genomics and Health [54,55] (GA4GH) through the organization of workflow execution challenges [56].

The containerization strategy is used by the GDC for their pipeline. They went further by investing effort into data harmonization, specifying a well-documented genomic data processing pipeline based on Genome Analysis ToolKit (GATK) Best Practices [57].

Ultimately, the goal of the processing step is to generate more easily usable files. In the case of genomic data, the main format is the variant call format [58] (VCF), and this format is supported by most of the major data-sharing platforms, such as the GDC or the International Cancer Genome Consortium (ICGC). In the context of precision oncology, this type of file typically contains data about somatic variants but is limited to single nucleotide variants (SNVs) or small indels (insertions–deletions). In contrast, there is not a clear standard for structural variants (SVs) and other genomic data, even though initiatives such as the Genomic Data Model and its associated query language, the GenoMetric Query Language [59] are proposing solutions to this issue.

The GATK Best Practices are one of the main standards for genomic file processing and are notably implemented by the GDC and are thus logical solutions. In precision oncology, VCF is the most interoperable format for somatic variants and containerization is a convenient way to ensure reusability, so these methods should be used as systematically as possible.

### Identification of variants

Standardizing the nomenclature of genomic alterations is needed to facilitate the findability of this type of data and thus should be implemented.

Large databases of somatic mutations, such as Catalogue Of Somatic Mutations In Cancer [60] (COSMIC) or dbSNP [61], have been built over the years. They include a large panel of these mutations, which are identified from major data-sharing initiatives, such as TARGET and TCGA, and are continuously updated from projects all over the world. Their internal identification mechanism could be considered as a good reference for identifying genomic alterations.

Nevertheless, they are based on previously observed mutations and many findings would in practice not be referenced, as a minority of researchers actually report their findings.

To solve this problem, a more systematic approach has been developed by the Human Genome Variation Society (HGVS), namely, the HGVS-nomenclature [62]. This approach makes it possible to give a unique identification to new variants and is supported by numerous initiatives, such as FHIR or the Database of Curated Mutations [63] (DoCM).

HGVS-nomenclature can thus be considered as the standard for naming genomic variants.

### Interpretations

The rationale for interpretations is to enrich somatic variations such as SNVs or SVs with clinically meaningful information, so they can be acted upon. Many resources already exist for that purpose [64].

Interpretations tend to make data less reusable as they depend on the current state of knowledge and local practices, and work has been done to mitigate this issue [65,66]. However, interpretations are useful for findability as adding interpretations to genomic data make them searchable by clinical significance and actionability. Interpretations can also be helpful for interoperability by defining standard terms. This is indeed the reason for some initiatives, for example in pharmacogenomics [46], which aim at interoperability of genomic-related data.

Numerous resources are available online [67,68]. Some of these resources clearly have a large impact, such as COSMIC or ClinVar [69], and to a lesser extent DoCM, and can be considered as primary references. The Ensembl Variant Effect Predictor [70] (VEP) tool is a convenient solution because it can annotate and provide a basic interpretation of variants.

Some initiatives are working on combining various available resources in a more comprehensive manner to provide an easy to integrate set of interpretations, including the Precision Medicine Knowledge Base [71], Precision Oncology Knowledge Base [72] (OncoKB) or Clinical Interpretations of Variants in Cancer [73]. While they are interesting resources, they could have some limitations regarding details and update frequency, due to their project-based financing structure.

At the clinical level, guidelines are being built to standardize the interpretation of sequence variants for better interoperability [74–76]. In Canada, the national initiative Canadian Open Genetics Repository [77] aims at standardizing genetic interpretations.

In parallel, some private companies are developing their own solutions using the aforementioned resources as well as internal curations. For example, Qiagen is developing its Clinical Insight [78] (QCI) platform with the goal of providing a better support and more systematic updates of the data within a proprietary knowledge base. Other solutions, such as VarSeq [79] or Alamut [80], are also available to enrich genomic data. Even large companies such as IBM have tried to enter the market with their Watson for Oncology [81]. Besides the cost of such solutions, it can be seen as problematic to rely on private companies to recommend potentially expensive treatments, even though this is already the case for companion diagnostics, which are now recommended for the development of targeted therapies [82].

While a commercial solution, such as QCI, appears to be the most reliable solution, its cost and potential conflict of interest may push towards the integration of alternative platforms from the academic world, such as VEP and OncoKB.

**Figure 2:**
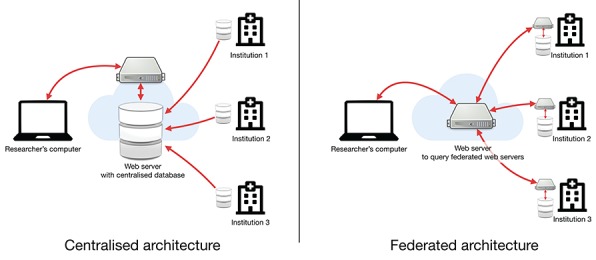
Comparison of centralized versus federated architectures. In the centralized architecture, each institution must upload their data to a centralized web server while in the federated architecture, data stay at their respective institutions, but each institution must implement an interface to make the data findable but not necessarily accessible.

## Data-sharing strategies

While data collection considerations are focused mostly on interoperability and reusability through common standards and harmonization, we still need to address data sharing to make data findable and accessible in practice.

### Network architecture: centralized versus federated

Data sharing requires an appropriate infrastructure, i.e. specifications of where data are stored and how they are accessed, which are central for findability and accessibility.

Conceptually, there are two approaches, the centralized or the federated approach [17] (Figure 2).

The centralized approach, followed by the GDC, is the classical solution and consists of gathering everything in one place. While this approach has advantages, such as guaranteeing a better harmonization of the data, it also faces major challenges. These challenges are mostly the drawbacks of the strict harmonization (rigidity), the sheer size of the project (inertia) and the massive quantity of data generated by HTS (transfer time).

The federated approach is a nimbler approach. Here, the goal is to make data easily searchable by defining an interface rather than a structure. One example is the Beacon Network developed by the GA4GH, which is an international consortium working at promoting and finding concrete solutions for data sharing.

The idea is that each centre stores its own data and makes them findable through an application programming interface (API). APIs make machine-to-machine communication possible and should be implemented in any modern platform [83,84].

In the federated approach, a centre can store any type of data according to its needs and still be a node in this network by implementing the API. The main objective of such a federated architecture is findability and less so accessibility. Therefore, the trade-off is that the stored data are not likely to be as interoperable as in the centralized scenario.

The federated approach implies that there is a search service, which can query the various nodes to aggregate the results. Every node must be responsible for the security of its own data. The level of security needed for storing and sharing sensitive patient data requires centres to employ experts to ensure that the platform is properly established and maintained, which can be challenging for smaller nodes.

Another federated approach is the BIOmedical and healthCAre Data Discovery Index Ecosystem's project, by the dataMED [85] prototype, which attempts to make data repositories searchable through a central search service. Here, each data set does not have to follow any specifications, but individual providers must make the data programmatically findable in a loosely defined format and the platform performs the mapping. FAIRness [39] is a clear goal of this platform, but the lack of coordination with integrated platforms can lead to more problematic long-term support and limitations concerning interoperability.

Due to the quantity, the heterogeneity and the potential specificities of data produced at the centre level, developing a data warehouse locally is becoming increasingly necessary, not only to share the data but also to handle the production and storage of such data. Adding an API to the local solution would be a straightforward endeavour and thus the federated approach promoted by the Beacon Network would be the most efficient option for large-scale data sharing. However, smaller research centres may not have enough resources to build such a platform on their own and may still need to join forces in a regionally centralized platform. Even the limited accessibility of data on federated solutions could be mitigated with existing distributed computation solutions [86].

### Access control: gate-keeper versus open access

Making data findable does not mean they are accessible, so the type of accessibility the data should follow to be FAIR should be defined while still complying with legal and ethical requirements.

There are mainly two approaches for accessibility: gate-keeper and open access. In the gate-keeper approach, data are not directly accessible and a request to access data is required, which must then go through an approval process. This is the safest approach of the two because it guarantees a certain level of control on who can access the data [87]. The approach is used by the European Genome-phenome Archive, which is a major repository of research data for biomedical sciences. This approach usually guarantees data of better quality and improves the FAIRness of the stored data, notably reusability, as there are often more validation steps involved. Nevertheless, it can be both complicated to implement as a data-sharing platform developer and cumbersome to use as a researcher.

In contrast, the open access approach implies that data are available without restriction and its goal is to build common genetic resources to foster research [88]. The main aim is accessibility, potentially to the detriment of other FAIR aspects. This type of access control is mostly used by de-identified research data repositories and reference projects, such as the 1000 Genomes Project [89] or the American Association for Cancer Research Genomics Evidence Neoplasia Information Exchange [90] project.

There is a trend to have a mix of the two approaches. For example, the GDC and the ICGC Data Portal restrict the access to some sensitive HTS data as there could be a risk for re-identifying participants [91] while leaving other data openly available.

The mixed approach followed by the GDC and ICGC seems to be the most pragmatic one, which allows one to keep more sensitive data under control while making less sensitive data easily accessible. However, this requires a large investment regarding platform development and can thus be prohibitive for individual research centres, which leaves the gate-keeper approach as the best option, to avoid legal and ethical complications.

## Conclusion

The aim of data sharing in precision oncology requires one to follow a set of principles to make the collected data FAIR. However, these principles can be expensive to implement, especially as there is no clear standard and a myriad of possible solutions exist.

There is a clear trend towards convergence, notably with the FHIR API supporting most of the listed recommendations, including genomic data [84], which could make them easily implemented in EHRs. This convergence is also driven by large initiatives such as the GDC, which tends to create *de facto* standards. Due to the sheer size of these types of projects, their requirements can handle different types of scenarios. In combination with well-established standards, such as ICD-O-3 classifications, MINSEQE, etc., the GDC specifications have defined relevant requirements for the implementation of the FAIR Data Principles in our own project.

While the FAIR principles define goals regarding data sharing, they do not consider ethics and costs, which are also key aspects when dealing with sensitive data, so the principles cannot stand alone.

While large repositories are needed for their structuring effects, they are expensive to maintain and not very flexible. Such initiatives should be handled at a national or international level because states and administrations have the resources to run them. The main purpose of such repositories is to be available for research centres for archiving their data.

Nevertheless, for more innovation-focused projects, the federated approach on top of a regionally centralized platform, which puts much less constraint on the data structure while still making the data findable, seems to be the more appropriate approach to make the data findable while allowing flexibility in the data structure.

### Perspectives

Progress in the understanding of cancer requires one to have access to larger and larger data sets and by allowing one to share data in a proper manner, future initiatives could leverage existing data to push science further ahead.

This study was initiated as a preparatory work for the development of a platform in precision oncology with data sharing in mind. More generally, by sharing our experience, we hope this work will facilitate the implementation of the FAIR Data Principles in national genome efforts and large oncological studies.

This study was nevertheless focused on genomic data, more specifically somatic mutations, alongside clinical data, which is a rather mature and well-documented approach. Similar work could be done in other omics fields, such as metabolomics or imaging.

## Notes

Key Points

- The FAIR Data Principles must be taken into account in the conception phase of the project
- Existing large-scale solutions, such as the GDC, can be considered *de facto* standards
- The implementation of existing standards, notably from WHO and HGVS, is mandatory for interoperability
- Containerization is a good way to ensure reusability
- Findability can be achieved through a federated infrastructure

## Notes

**Charles Vesteghem** is doing a PhD at the Department of Clinical Medicine, Aalborg University on precision oncology. He holds a double MSc in engineering with a specialization in biomedical engineering and worked in data-driven start-ups before joining the Department of Haematology, Aalborg University Hospital as a data manager/solution architect.

## Notes

**Rasmus Froberg Brøndum** holds an MSc in Statistics, and a PhD in quantitative genetics. He currently works as a senior bioinformatician at Aalborg University Hospital, focusing on data-driven research in haematological malignancies.

## Notes

**Mads Sønderkær** holds an MSc in biotechnology, and a PhD in bioinformatics. He currently works as a senior bioinformatician at Aalborg University Hospital, focusing on data-driven research in haematological malignancies.

## Notes

**Mia Sommer** is doing a PHD in health science at the Department of Hematology, Aalborg University Hospital and Department of Clinical Medicine, Aalborg University. She holds a masters degree in health science and an bachelor degree in nursing.

## Notes

**Alexander Schmitz** holds a doctoral degree (dr. rer. nat.) in genetics and molecular biology. He works as key operator/flow cytometry facility manager at the Department of Haematology, Aalborg University Hospital. His research is focusing on minimal residual disease in B-cell malignancies like multiple myeloma, and phenotypic and functional heterogeneity among cancer cells.

## Notes

**Julie Støve Bødker** holds a Msc and PhD in Molecular Biology and is a senior research scientist at Department of Hematology, Aalborg University Hospital. She maintains and develops our biobank of biological samples, sequence selected samples and interprets the detected tumor specific DNA variants in the context of personalized medicine.

## Notes

**Karen Dybkær** is Professor in Molecular Hematology, Department of Clinical Medicine, Aalborg University/Department of Hematology, Aalborg University Hospital. She has an experimental background and a special interest in deciphering the role of cellular differentiation on tumor development and individual response to chemotherapy.

## Notes

**Tarec Christoffer El-Galaly** is a haematologist at Aalborg University Hospital and professor in clinical hematology with focus on real world evidence integration in decision making. He has led national and international studies and collaborations on outcomes research, therapeutic interventions and advanced prognostic modelling based on real world data in lymphoma.

## Notes

**Martin Bøgsted** is Professor in Bioinformatics and Statistics at Department of Clinical Medicine at Aalborg University and Senior Statistician at Department of Haematology, Aalborg University and holds a MSc degree in mathematics and statistics as well as a PhD degree in statistics. His main interest is in statistical issues related to translating high throughput biological and big clinical data findings to clinical practice.
